# *Dendrobium Officinale* Polysaccharide Inhibits Ferroptosis and Ameliorates Cerebral Ischemia–Reperfusion Injury: Involvement of the Nrf2/GPX4 Signaling Pathway

**DOI:** 10.1007/s12035-026-06208-w

**Published:** 2026-10-05

**Authors:** Xiaoxiao Li, Wenxin Xia, Caiyu Yang, Chenchen Xu, Xin Wang, Jianjian Dong, Yongsheng Han

**Affiliations:** 1 Wannan Medical University, Wuhu, Anhui China; 2 Anhui University of Chinese Medicine, Hefei, China; 3 Institute of Neurology, Anhui University of Chinese Medicine, Hefei, China; 4 Center for Xin’an Medicine and Modernization of Traditional Chinese Medicine of IHM, Anhui University of Chinese Medicine, Hefei, China

**Keywords:** Cerebral ischemia–reperfusion injury, *Dendrobium officinale* polysaccharide, Ferroptosis, Nrf2/GPX4 signaling pathway

## Abstract

Ferroptosis, a critical pathological mechanism in ischemic stroke (IS), is activated during the early phase of cerebral ischemia–reperfusion injury (CI/RI). *Dendrobium officinale* polysaccharide (DOP), the primary bioactive component of *Dendrobium officinale*, exhibits diverse pharmacological activities, including antioxidant, anti-inflammatory, and immunomodulatory effects. DOP has been suggested to exert neuroprotective effects by inhibiting ferroptosis; however, its specific role and underlying mechanisms in IS-related ferroptosis remain unclear. In this study, in vivo and in vitro CI/RI models were established using middle cerebral artery occlusion/reperfusion (MCAO/R) and oxygen–glucose deprivation/reoxygenation (OGD/R), respectively. DOP was administered under a paradigm combining long-term pretreatment and short-term post-ischemic intervention, and its neuroprotective effects were evaluated. In MCAO/R mice, DOP significantly alleviated neurological deficits, reduced the cerebral infarct volume, and mitigated neuronal damage. These effects were associated with decreased Fe^2^⁺ accumulation and lipid peroxidation, ameliorated mitochondrial impairment, and upregulated the expression of key ferroptosis-related proteins, including nuclear factor erythroid 2-related factor 2 (Nrf2) and glutathione peroxidase 4 (GPX4). Consistent with in vivo findings, DOP enhanced the viability of HT-22 cells subjected to OGD/R, decreased reactive oxygen species, and restored the mitochondrial membrane potential, accompanied by increased Nrf2 and GPX4 expression. Notably, knockdown of Nrf2 attenuated GPX4 expression and diminished the protective effects of DOP. Collectively, these findings suggest that DOP exerts neuroprotective effects against CI/RI, at least in part, by inhibiting ferroptosis through the Nrf2/GPX4 signaling pathway, supporting its potential role in preventive and early-stage intervention strategies for ischemic stroke.

## Introduction

Stroke is the second leading cause of death globally and the third leading cause of combined mortality and disability [1], representing a major contributor to the global disease burden [2]. Ischemic stroke (IS), the most prevalent form of stroke, is a neurological disorder characterized by interrupted cerebral blood flow due to vascular occlusion, which results in ischemic and hypoxic damage of brain tissue [3]. Approximately 7.8 million new cases of IS are reported annually worldwide, accounting for 62.4% of all stroke cases [4]. Timely recanalization of occluded blood vessels remains the primary therapeutic objective for IS. While advancements in intravenous thrombolysis and mechanical thrombectomy have improved outcomes for some patients [5], rapid reperfusion of ischemic tissue often triggers a cascade of pathological responses—including a burst of reactive oxygen species (ROS), activation of the microglia and astrocytes, and infiltration of inflammatory mediators—resulting in the mutual amplification of damage response pathways and exacerbation of neuronal death, thereby aggravating cerebral ischemia–reperfusion injury (CI/RI) [6].

Ferroptosis, a non-apoptotic form of cell death characterized by iron-dependent membrane lipid peroxidation, is defined by dysregulated iron homeostasis and the accumulation of lipid peroxides [7]. Increasing evidence indicates that ferroptosis plays a critical role in the pathophysiology of IS; therefore, the inhibition of ferroptosis is a promising therapeutic strategy [8–10]. Recent studies have confirmed that ferroptosis is activated during the early phase of CI/RI, and ferroptosis inhibitors can partially mitigate reperfusion injury [11]. Nuclear factor erythroid 2-related factor 2 (Nrf2) serves as a master regulator of cellular antioxidant responses and a key modulator of ferroptosis. Nrf2 target genes encode various antioxidant enzymes and iron metabolism-related proteins, including glutathione peroxidase 4 (GPX4) [12]. As a pivotal antioxidant enzyme and central inhibitor of ferroptosis, the GPX4 protein reduces toxic lipid peroxides to non-toxic lipid alcohols [13]. Numerous studies have demonstrated that activation of the Nrf2/GPX4 signaling pathway reduces neuronal susceptibility to ferroptosis [14, 15].

*Dendrobium*, a medicinal orchid genus native to China, has long been used in traditional Chinese medicine. *Dendrobium officinale* is a particularly valued species, and its polysaccharide fraction, *Dendrobium officinale* polysaccharide (DOP), exhibits diverse biological activities, including antioxidant, anti-inflammatory, immunomodulatory, antitumor, and hypoglycemic effects [16]. Previous studies have shown that polysaccharides derived from *Dendrobium nobile* can alleviate neurological injury and promote functional recovery in models of cerebral ischemia/reperfusion, vascular dementia, and spinal cord injury, partly through suppression of ferroptosis [17–19]. However, these findings were obtained using D. nobile polysaccharides and therefore cannot be directly extrapolated to D. officinale polysaccharide. In contrast, studies of DOP have mainly focused on its antioxidant and anti-inflammatory activities, including activation of the Nrf2 signaling pathway [20–22], whereas its role in ferroptosis during CI/RI remains largely unexplored. Based on these observations, we hypothesized that DOP may attenuate CI/RI, at least in part, by suppressing ferroptosis, potentially involving the Nrf2/GPX4 signaling pathway.

To test this hypothesis, we employed a mouse model of middle cerebral artery occlusion/reperfusion (MCAO/R) and an in vitro oxygen–glucose deprivation/reoxygenation (OGD/R) model to systematically investigate the neuroprotective effects of DOP. By integrating multiple molecular and cellular approaches, we aimed to elucidate the underlying mechanisms of DOP in the context of CI/RI. This study provides mechanistic insights into the protective actions of DOP and supports its potential relevance in preventive and early-stage intervention strategies for IS.

## Materials and Methods

### Animals and Establishing the MCAO/R Model

Adult male C57BL/6 mice (6-week-old, 25–30 g) were housed under standard laboratory conditions at a temperature of 24–26 ℃ and relative humidity of 50–60%, with a 12 h light/dark cycle and free access to food and water. All experimental procedures were conducted in accordance with the National Institutes of Health Guidelines for the Care and Use of Laboratory Animals and approved by the Animal Ethics Committee of Anhui University of Chinese Medicine (Approval No. AHUCM-mouse-2023131).

The CI/RI model was induced using the MCAO/R technique [23]. Briefly, the left MCA was selected as the occlusion side. Mice were anesthetized via intraperitoneal injection of 3% sodium pentobarbital (30 mg/kg) and placed in a supine position on a heating pad to maintain body temperature at 37.0 ± 0.5 °C. After disinfecting the skin on the neck with iodine, a 1 cm incision was made using surgical scissors. The neck muscles were bluntly dissected to expose the common carotid artery (CCA), external carotid artery (ECA), and internal carotid artery (ICA). The proximal CCA and ECA were ligated, and the ICA was temporarily clamped. A small cut was made in the CCA, and a silicone-coated monofilament (1220-A5, Beijing Xinong Technology Co., Ltd, China) was inserted from the CCA into the ICA until slight resistance was felt, approximately 10 ± 1 mm beyond the carotid bifurcation. After 1 h of occlusion, the filament was withdrawn to allow reperfusion, and the incision was sutured layer by layer.

### Grouping and Drug Administration

Following 1 week of acclimatization, the mice were randomly assigned into six groups: normal control (NC), sham (Sham), cerebral ischemia/reperfusion model (MCAO/R), MCAO/R plus low-dose DOP (MCAO/R + DOP-L, 100 mg/kg), MCAO/R plus high-dose DOP (MCAO/R + DOP-H, 200 mg/kg) [17], and MCAO/R plus Ferrostatin-1 (MCAO/R + Fer-1, 10 mg/kg) [24]. The sample size was determined based on commonly used group sizes in MCAO/R studies and preliminary experimental results showing stable trends in outcome measures [25, 26].

The NC group received no treatment. Mice in the Sham group underwent the same surgical procedures as the MCAO/R group except that the carotid arteries were exposed without insertion of the filament. Mice in all remaining groups underwent MCAO/R surgery. The DOP (Catalog No. S35546, purity 90%, Shanghai Yuanye Bio-Technology Co., Ltd, China) was dissolved in physiological saline and administered via oral gavage once daily. Mice in the DOP treatment groups received DOP for 30 consecutive days before MCAO/R induction and continuously for 3 days after reperfusion. To control for the influence of long-term gavage procedures, mice in the NC and MCAO/R groups received an equal volume of saline according to the same schedule. Ferrostatin-1 (Fer-1; S7243, Selleck, USA), a specific ferroptosis inhibitor, was dissolved according to the manufacturer’s instructions and administered intraperitoneally at a dose of 10 mg/kg at 24 h and 2 h before MCAO/R surgery. The MCAO/R + Fer-1 group served as a positive control for ferroptosis inhibition to enable mechanistic comparison with DOP treatment and to better evaluate the contribution of ferroptosis regulation to DOP-mediated neuroprotection following cerebral ischemia/reperfusion injury.

### Behavioral Assessments

All behavioral tests were performed 24 h after model induction using a double-blind design to minimize bias, with experimenters blinded to group assignments.

#### Neurological Function Scoring

Neurological deficits were evaluated according to the Zea Longa scoring system [27]: 0 points, no neurological deficit; 1 point, failure to fully extend the left forelimb; 2 points, circling to the right during movement; 3 points, leaning or falling to the right; 4 points, unable to walk spontaneously or loss of consciousness.

#### Rotarod Test

Motor coordination and endurance were assessed using a rotarod apparatus. Mice underwent three trials per day for 3 consecutive days, with the first 2 days serving as acclimatization training. Each trial accelerated from 5 r/min to 40 r/min over 5 min, with a 30-min inter-trial interval. The latency to fall or a maximum duration of 300 s was recorded. The average latency across three trials on the test day was used for the analysis.

#### Balance Beam Test (BBT)

Motor coordination and balance were evaluated using a narrow beam (100 cm long, 2 cm wide, 50 cm above the ground). A dark box containing home-cage bedding was placed at the end of the beam as a target. After 2 days of acclimatization, each mouse was tested for traversal time, number of falls, and postural adjustments. Each trial was limited to 2 min. Mice scoring 1 point in two consecutive trials were not retested. Motor performance was evaluated using the Feeney scoring criteria [28].

### TTC Staining

Mice were euthanized with 3% sodium pentobarbital and decapitated, followed by brain extraction. Coronary brain sections were incubated with 2,3,5-triphenyltetrazolium chloride (TTC) staining solution (G1017, ServiceBio, Wuhan, China) at 37 °C for 30 min, protected from light. After color development, the reaction was terminated with fixative. Tissues were stored in the dark and imaged. The infarct volume was analyzed and quantified using ImageJ software.

### FJB Staining

Paraffin-embedded brain sections were dewaxed and hydrated. After circling with a histopen, a working solution of Fluoro-Jade B (FJB) was prepared at a 1:400 dilution using 50% glacial acetic acid as the solvent. The diluted FJB green fluorescent probe was applied to the sections and incubated overnight at 4 °C. Nuclei were then stained with DAPI for 8 min and rinsed with pure water. After drying, sections were transparentized with xylene for 1 min and mounted with quick-drying mounting medium. Images were acquired using an upright fluorescence microscope (Nikon Eclipse C1, Japan). FJB-positive cells were quantified with ImageJ.

### Assessment of Cerebral Blood Flow and Vascular Signals

Cerebral blood flow (CBF) and vascular signals were assessed using laser speckle contrast imaging (LSCI) and super-resolution ultrasound imaging.

For LSCI, CBF was monitored in real time using a laser speckle contrast imaging system (RFLSI ZW, RWD Life Science). Mice were anesthetized with 4% isoflurane for induction and maintained at 1.5–2% during imaging, with body temperature kept at 37 °C. Following a midline scalp incision, the skull was exposed and kept moist with physiological saline. Speckle images were continuously acquired and averaged over 250 frames (~ 5 s). Representative perfusion images were obtained from the cortical region within the territory of the left middle cerebral artery. For ultrasound imaging, cerebral vascular signals were evaluated using a small animal super-resolution ultrasound system (ULTIMUS 9LAB, VINNO, Suzhou, China). Mice were prepared under the same conditions as described above. A high-frequency probe was positioned over the cranial surface, and microvascular imaging was performed to visualize cortical vessels. Representative images were acquired from multiple fields per animal to assess changes in vascular distribution and blood flow signals.

### Transmission Electron Microscopy (TEM)

The mitochondrial ultrastructure in the ischemic cortex was observed using TEM (HT7800/HT7700, Hitachi). Ischemic cortical tissues were dissected into 1 mm^3^ pieces and fixed in 1% osmium tetroxide at room temperature in the dark. Samples were then dehydrated, embedded, polymerized, sectioned, and stained with uranyl acetate and lead citrate. Grids were examined under TEM, and images were acquired for analysis.

### Cell Culture

The mouse hippocampal neuronal cell line HT-22 was obtained from the Chinese Academy of Sciences Cell Bank (Shanghai, China). Cells were cultured in high-glucose DMEM medium supplemented with 10% fetal bovine serum (FBS), 1% streptomycin, and 1% penicillin at 37 °C under 5% CO₂. The medium was replaced every 1–2 days. Upon reaching approximately 90% confluence, cells in the logarithmic growth phase were sub-cultured for subsequent experiments.

### OGD/R Modeling and DOP Treatment

An in vitro ischemia–reperfusion (I/R) model was established using the OGD/R method. Briefly, after two washes with phosphate-buffered saline (PBS), cells were cultured in glucose- and serum-free DMEM medium and placed in a tri-gas incubator (37 °C, 95% N₂, 5% CO₂) for 2 h. The medium was then replaced with complete high-glucose DMEM containing 10% FBS, followed by incubation under normoxic conditions (37 °C, 5% CO₂) for 24 h. Control cells were maintained under normal culture conditions. DOP pretreatment was administered 24 h prior to OGD/R induction.

### siRNA Transfection

HT-22 cells were seeded into 6-well plates containing antibiotic-free high-glucose DMEM supplemented with 10% FBS and cultured until they reached 30–50% confluence. The transfection was performed using the RNAFit transfection reagent (Hanbio, Shanghai, China) according to the manufacturer’s instructions. A mixture of 1.8 mL medium and 200 μL transfection solution was added to each well. Nrf2-targeting siRNA duplexes (sequences: mm-NRF2-S: 5′-CUCGCAUUGAUCCGAGAUAdTdT-3′; mm-NRF2-A: 5′-UAUCUCGGAUCAAUGCGAGdTdT-3′) were synthesized by Hanbio. After 48 h of transfection, cells were harvested for subsequent experiments. Transfection efficiency was evaluated using real-time quantitative polymerase chain reaction (RT-qPCR).

### Cell Viability Assay

Cell viability was assessed using the Cell Counting Kit-8 (CCK-8, C0038, Beyotime, Shanghai, China) according to the manufacturer’s instructions. Results were expressed as a percentage of the viability in the control or model group.

### TUNEL Staining

Terminal deoxynucleotidyl transferase-mediated dUTP nick end labeling (TUNEL) staining was performed using a commercial kit (G1501, ServiceBio). Briefly, cells were fixed with 4% paraformaldehyde for 20 min at room temperature, permeabilized, and then incubated in equilibration buffer. The TdT reaction mixture was applied for 1 h at 37 °C. Nuclei were stained with DAPI. After sealing with anti-fade mounting medium, the cells were imaged under a fluorescence microscope. The percentage of TUNEL-positive cells was quantified using ImageJ.

### ROS Detection

Intracellular ROS levels were measured using a detection kit (G1706, ServiceBio). Cells were incubated with DCFH-DA working solution for 30 min at 37 °C, protected from light. After washing with PBS, nuclei were stained using an anti-fade mounting medium containing DAPI (G1407, ServiceBio). Fluorescence images were captured under a microscope.

### Mitochondrial Membrane Potential (MMP) Assay

Changes in the MMP were evaluated using the JC-1 assay kit (C2006, Beyotime). Cells were incubated with the JC-1 working solution for 20 min at 37 °C, washed twice with assay buffer, and then mounted. Fluorescence was immediately imaged using a fluorescence microscope.

### ATP Assay

Intracellular ATP levels were measured using an enhanced ATP assay kit (S0027, Beyotime) according to the manufacturer’s instructions. Briefly, cells were lysed with ATP lysis buffer on ice and centrifuged at 12,000 × g for 5 min at 4 °C. The supernatant was collected, and ATP levels were determined using a luciferase-based detection system. Luminescence was measured using a microplate reader. ATP levels were expressed as relative values normalized to the control group.

### RT-qPCR

Total RNA was extracted using a Tissue/Cell RNA Rapid Extraction Kit (AC0202-B, SparkJade, Shangdong, China). cDNA was synthesized with the SPARKscript Ⅱ RT Plus Kit (with a gDNA Eraser, AG0304-B, SparkJade). Quantitative PCR amplification was performed using 2 × SYBR Green qPCR Mix (with ROX, AH0104-B, SparkJade) on an ABI 7500 Real-Time PCR System under the following conditions: initial denaturation at 94 °C for 2–3 min; 40 cycles of denaturation at 94 °C for 10–20 s, annealing at 55–60 °C for 10–20 s, and extension at 72 °C for 20–30 s. Relative gene expression was calculated using the 2^^(−ΔΔCt)^ method, with β-actin as the internal control. Primer sequences are listed in Table 1.

**Table 1:** Primer sequences

Gene	Forward (5'−3')	Reverse (5'−3')
β-actin	GGCTGTATTCCCCTCCATCG	CCAGTTGGTAACAATGCCATGT
Nrf2	TCTTGGAGTAAGTCGAGAAGTGT	GTTGAACTGAGCGAAAAAGGC
GPX4	GATGGAGCCCATTCCTGAACC	CCCTGTACTTATCCAGGCAGA
Tf	TACCATCAGGGCACAGCAGC	GGCATCAGACTCCAGCATCA
ACSL4	CTCACCATTATATTGCTGCCTGT	TCTCTTTGCCATAGCGTTTTTCT

### Western Blot

Total protein was extracted from tissues or cells using RIPA lysis buffer (R0020, Solarbio, Beijing, China) containing phenylmethylsulfonyl fluoride (PMSF; FJP0100, Solarbio). The protein concentration was determined with a bicinchoninic acid (BCA) assay. Approximately 30 μg of protein per sample was separated by sodium dodecyl sulfate–polyacrylamide gel electrophoresis (SDS–PAGE) and transferred to polyvinylidene difluoride membrane (PVDF) membranes. After blocking with 5% non-fat milk, membranes were incubated overnight at 4 °C with the following primary antibodies: Nrf2 (380,773, 1:1000, Zen-Bio, Chengdu, China), GPX4 (381,958, 1:1000, Zen-Bio), Tf (A19130, 1:1000, ABclonal, Wuhan, China), ACSL4 (A20414, 1:1000, ABclonal), GAPDH (GB15004-100, 1:5000, ServiceBio), and β-tubulin (GB380628, 1:5000, ServiceBio). Membranes were then washed with tris-buffered saline with tween (TBST) and incubated with horseradish peroxidase (HRP)-conjugated secondary antibodies at room temperature. Protein signals were visualized using an enhanced chemiluminescence (ECL) substrate (G2161, ServiceBio) and imaged with a Tanon Chemiluminescence Imaging System (China). Band intensities were quantified using ImageJ. Western blot analysis was used as the primary method for quantitative assessment of protein expression in this study.

### Immunofluorescence

Paraffin-embedded brain sections were dewaxed and rehydrated. Antigen retrieval was performed by heating in citrate buffer (pH 6.0) at 95 °C for 8 min. Sections were blocked with 3% bovine serum albumin (BSA) for 30 min at room temperature and incubated overnight at 4 °C with primary antibodies against Nrf2 (380,773, 1:1000, Zen-Bio), ACSL4 (A20414, 1:1000, ABclonal), GPX4 (381,958, 1:1000, Zen-Bio), 4-HNE (AB46545, 1:1000, Abcam, Cambridge, MA, USA), and NeuN (GB11138, 1:1000, ServiceBio). After washing, sections were incubated with fluorescence-conjugated secondary antibodies for 50 min at room temperature, protected from the light. Nuclei were stained with DAPI, and autofluorescence was quenched. Sections were mounted with anti-fade mounting medium and imaged under a fluorescence microscope. Immunofluorescence staining was primarily used to assess the cellular localization and neuron-specific expression patterns of target proteins. Semi-quantitative analysis of fluorescence intensity was performed using ImageJ and interpreted in conjunction with Western blot results.

### Detection of SOD, MDA, GSH, GSSG, Fe^2⁺^ and GPX4 Activity

Levels of superoxide dismutase (SOD, BC0175, Solarbio), malondialdehyde (MDA, BC0025, Solarbio), reduced glutathione (GSH, BC1175, Solarbio), oxidized glutathione (GSSG, BC1185, Solarbio), Fe^2^⁺ (BC5415, Solarbio), and GPX4 activity (BC6260, Solarbio) in ischemic brain tissues were measured using commercial assay kits according to the manufacturers’ instructions.

### Statistical Analysis

Data were analyzed and visualized using GraphPad Prism 9 (GraphPad Software, La Jolla, CA, USA). All quantitative results are expressed as the mean ± standard deviation (SD). Comparisons between two groups conforming to a normal distribution were performed using an unpaired two-tailed Student’s t-test. For comparisons among multiple groups with a normal distribution and homogeneity of variance, the one-way ANOVA followed by Tukey’s post hoc test was applied. When normality was met but variances were unequal, Brown–Forsythe and Welch ANOVA tests were used. For data that did not follow a normal distribution, the Kruskal–Wallis test was employed. *P* < 0.05 was considered statistically significant. Unless otherwise indicated, n represents independent biological replicates for in vitro experiments and independent animals for in vivo experiments; technical replicates were not considered independent samples.

## Results

### DOP Attenuates Cerebral Injury in the MCAO/R Model Mice

**Fig. 1:**
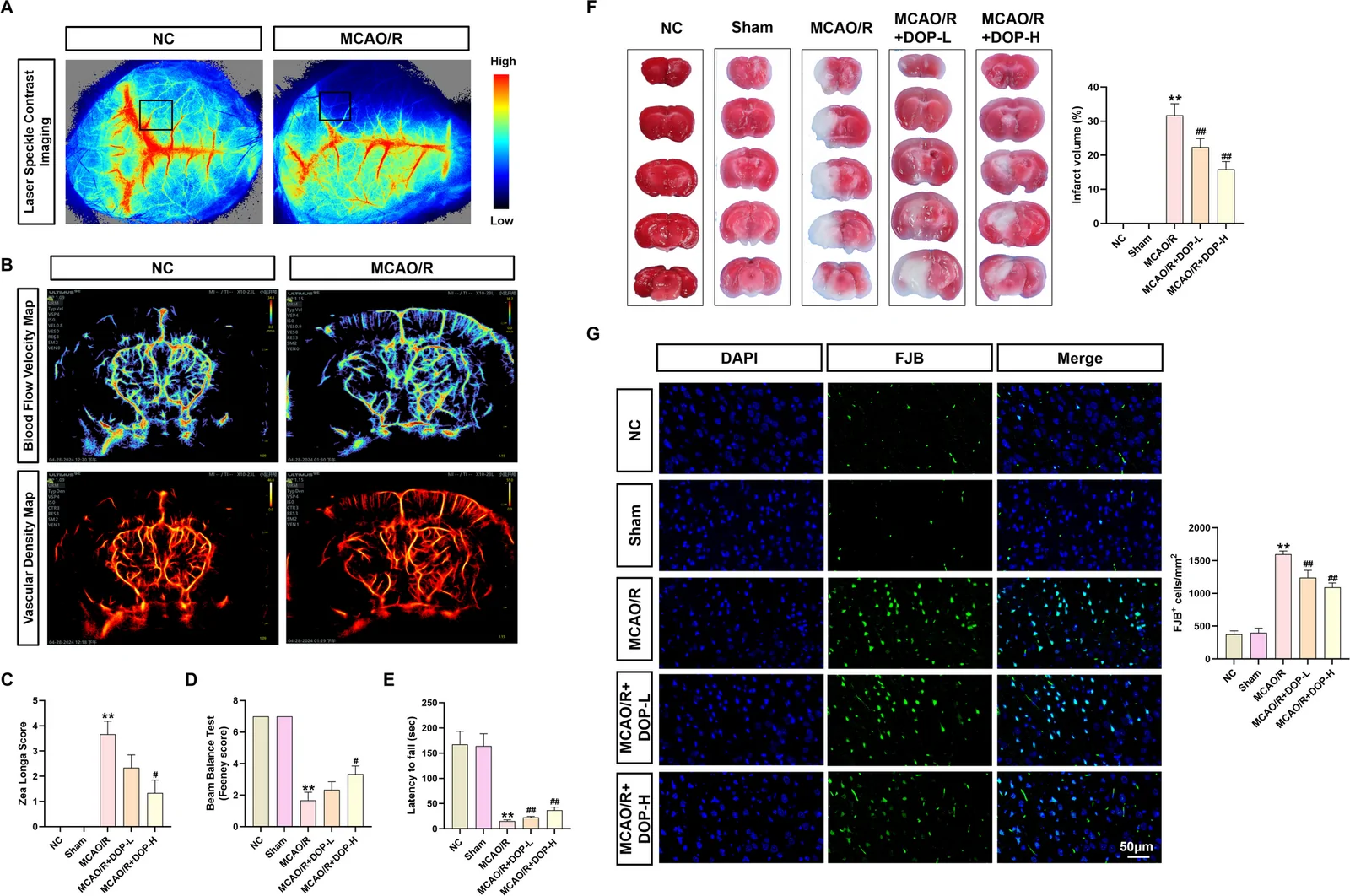
DOP attenuates cerebral injury in MCAO/R mice. **A** Representative laser speckle contrast imaging (LSCI) images showing cortical cerebral blood flow in the NC and MCAO/R groups, confirming cerebral ischemia after MCAO/R. *n* = 6/group. **B** Representative super-resolution ultrasound images showing cortical microvascular perfusion in the NC and MCAO/R groups, confirming impaired cerebral perfusion after MCAO/R. *n* = 6/group. **C** Zea Longa neurological scores. *n* = 6/group. **D** Balance beam test (BBT). *n* = 6/group. **E** Rotarod test. *n* = 6/group. **F** Representative TTC-stained brain sections and quantification of cerebral infarct volume. *n* = 6/group. **G** Representative images of FJB staining in the left cerebral cortex. Scale bar = 50 μm, *n* = 6/group. ^**^P < 0.01 vs. NC group; ^#^*P* < 0.05, ^##^*P* < 0.01 vs. MCAO/R group

Representative laser speckle contrast imaging (LSCI) images were used to assess cortical cerebral blood flow (CBF). Compared with the NC group, mice subjected to MCAO/R showed a marked reduction in cortical perfusion signals, particularly within the territory of the left middle cerebral artery (Fig. 1A). Consistently, super-resolution ultrasound imaging revealed reduced cortical microvascular signals in the MCAO/R group (Fig. 1B). These findings were primarily used to confirm impaired cerebral perfusion and successful establishment of the MCAO/R model.

To exclude the potential influence of surgical procedures, a sham-operated group was included. No significant differences were observed between the NC and Sham groups in behavioral assessments, including the Zea Longa score, BBT, and rotarod test (Fig. 1C–E). In contrast, the MCAO/R group showed severe neurological deficits, as indicated by increased Zea Longa scores, reduced Feeney scores, and shorter rotarod latencies. DOP treatment markedly improved neurological performance compared with the MCAO/R group. TTC staining demonstrated no evident infarction in the NC and Sham groups, whereas a substantial infarct area was observed in the MCAO/R group, which was significantly reduced following DOP administration (Fig. 1F). Similarly, FJB staining revealed minimal neuronal degeneration in the NC and Sham groups, while the number of FJB-positive cells was markedly increased after MCAO/R and attenuated by DOP treatment (Fig. 1G). Collectively, these results indicate that DOP improves neurological function, reduces infarct volume, and alleviates neuronal damage in MCAO/R mice.

### DOP Inhibits Ferroptosis in the MCAO/R Model Mice

**Fig. 2:**
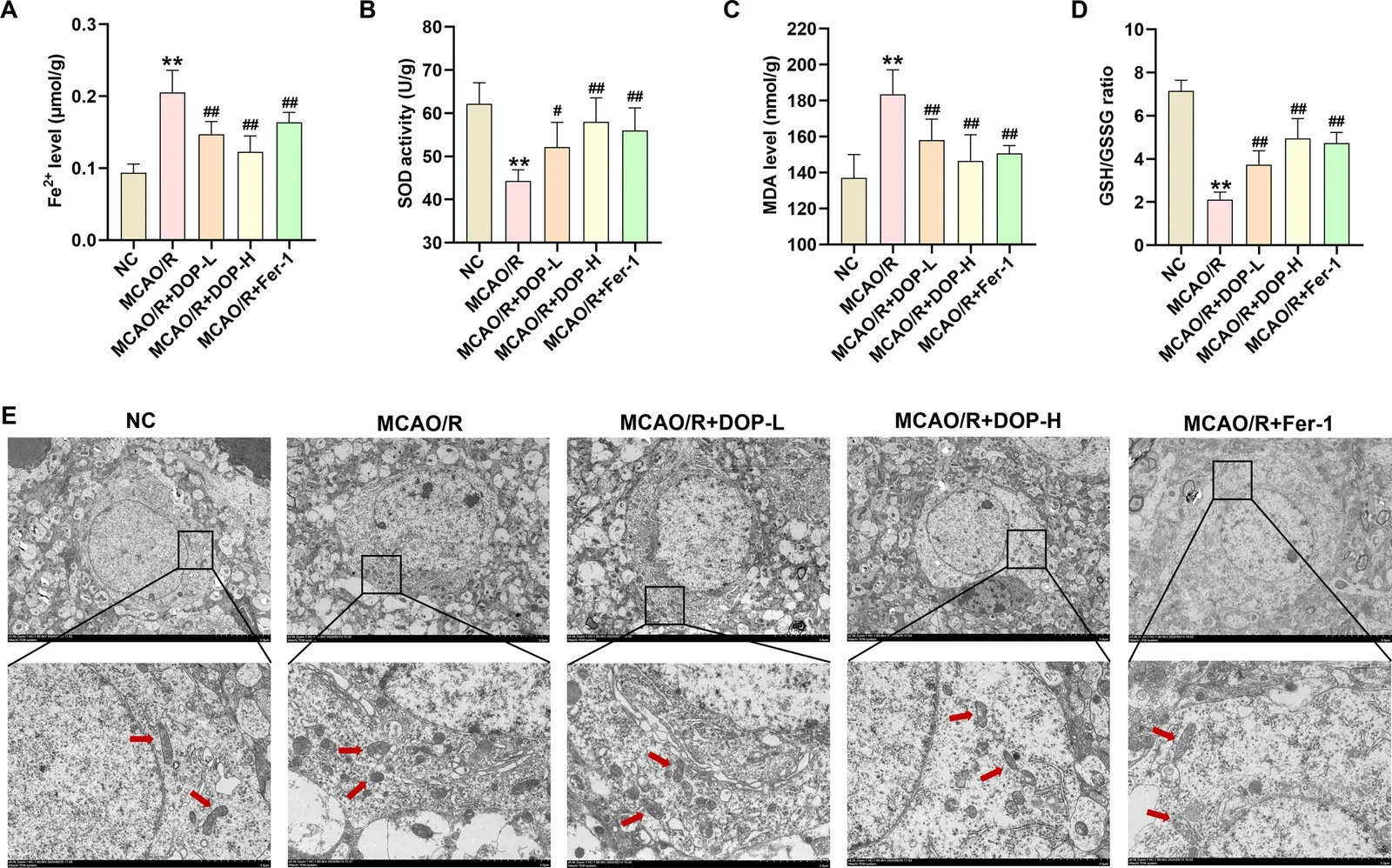
DOP inhibits ferroptosis in the MCAO/R model mice. **A** Fe^2^⁺ content. *n* = 6/group. **B** SOD activity. *n* = 6/group. **C** MDA levels. *n* = 6/group. **D** GSH/GSSG ratio. *n* = 6/group. **E** Representative TEM images of neuronal mitochondria in the ischemic cortex. Red arrows indicate mitochondria. Scale bars = 5 μm/2 μm. *n* = 6/group. ^**^P < 0.01 vs. NC group; ^#^*P* < 0.05, ^##^*P* < 0.01 vs. MCAO/R group

**Fig. 3:**
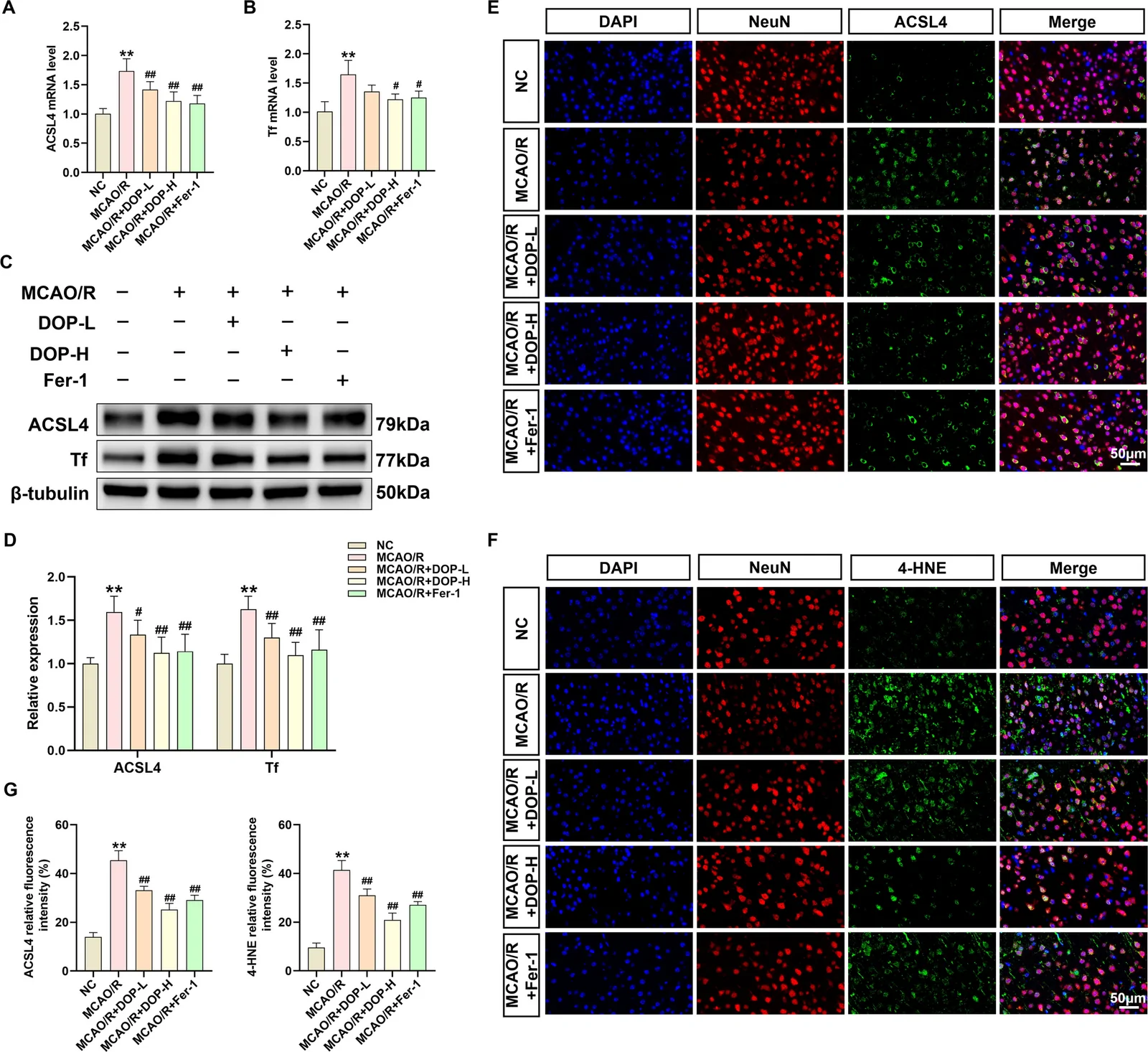
DOP regulates the expression of ferroptosis-related molecules in the MCAO/R model mice. **A** *ACSL4* mRNA expression measured by RT-qPCR. *n* = 6/group. **B** *Tf* mRNA expression measured by RT-qPCR. *n* = 6/group. **C** Representative Western blot images showing protein expression levels of ACSL4 and Tf. *n* = 6/group. **D** Quantitative analysis of ACSL4 and Tf protein expression. *n* = 6/group. **E** Immunofluorescence staining of neurons (red) and ACSL4 (green) in the ischemic cerebral cortex. Scale bar = 50 μm. *n* = 6/group. **F** Immunofluorescence staining of neurons (red) and 4-HNE (green) in the ischemic cerebral cortex. Scale bar = 50 μm. *n* = 6/group. **G** Quantitative analysis of ACSL4 and 4-HNE fluorescence signals. *n* = 6/group. ^**^P < 0.01 vs. NC group; ^#^*P* < 0.05, ^##^*P* < 0.01 vs. MCAO/R group

Disrupted iron homeostasis and accumulated lipid peroxidation are established pathological hallmarks of ferroptosis [29]. To investigate whether DOP modulates ferroptosis following CI/RI, we measured Fe^2^⁺ levels and lipid peroxidation markers in the ischemic cerebral hemispheres (Fig. 2A–D). The MCAO/R group exhibited elevated Fe^2^⁺ content, decreased SOD activity, increased MDA levels, and a reduced GSH/GSSG ratio. Both DOP treatment and the ferroptosis inhibitor Fer-1 markedly reversed these alterations, as evidenced by reduced Fe^2^⁺ accumulation, enhanced SOD activity, decreased MDA levels, and restoration of the GSH/GSSG ratio, indicating effective suppression of ferroptosis processes. Mitochondrial ultrastructural damage represents a key morphological indicator of ferroptosis [30]. TEM revealed shrunken mitochondria with condensed membranes, outer membrane rupture, and loss of cristae in MCAO/R mice. These pathological changes were markedly ameliorated following DOP or Fer-1 administration, further supporting their inhibitory effects on ferroptosis (Fig. 2E). We next examined key ferroptosis biomarkers, including acyl-CoA synthetase long-chain family member 4 (ACSL4), 4-hydroxynonenal (4-HNE), and transferrin (Tf) [31]. ACSL4 esterifies free polyunsaturated fatty acids (PUFAs) into phospholipids, promoting membrane lipid peroxidation and increasing cellular susceptibility to ferroptosis. 4-HNE is a lipid peroxidation end-product, and Tf facilitates cellular iron uptake [32]. RT-qPCR and Western blot analyses demonstrated upregulation of ACSL4 and Tf expression in MCAO/R mice at both the transcriptional and protein levels, which was suppressed by DOP and Fer-1 treatments (Fig. 3A–D). Consistently, immunofluorescence staining revealed increased ACSL4 and 4-HNE signals in the MCAO/R group, primarily localized in neurons, which were attenuated following DOP or Fer-1 intervention (Fig. 3E–G). These findings indicate that MCAO/R induces neuronal ferroptosis, characterized by iron dysregulation, lipid peroxidation, and mitochondrial impairment. Both DOP and Fer-1 effectively mitigated these pathological changes, suggesting a protective role against ferroptosis.

### DOP Promotes Nrf2 Nuclear Translocation and Restores Neuronal GPX4 Expression in the MCAO/R Model Mice

**Fig. 4:**
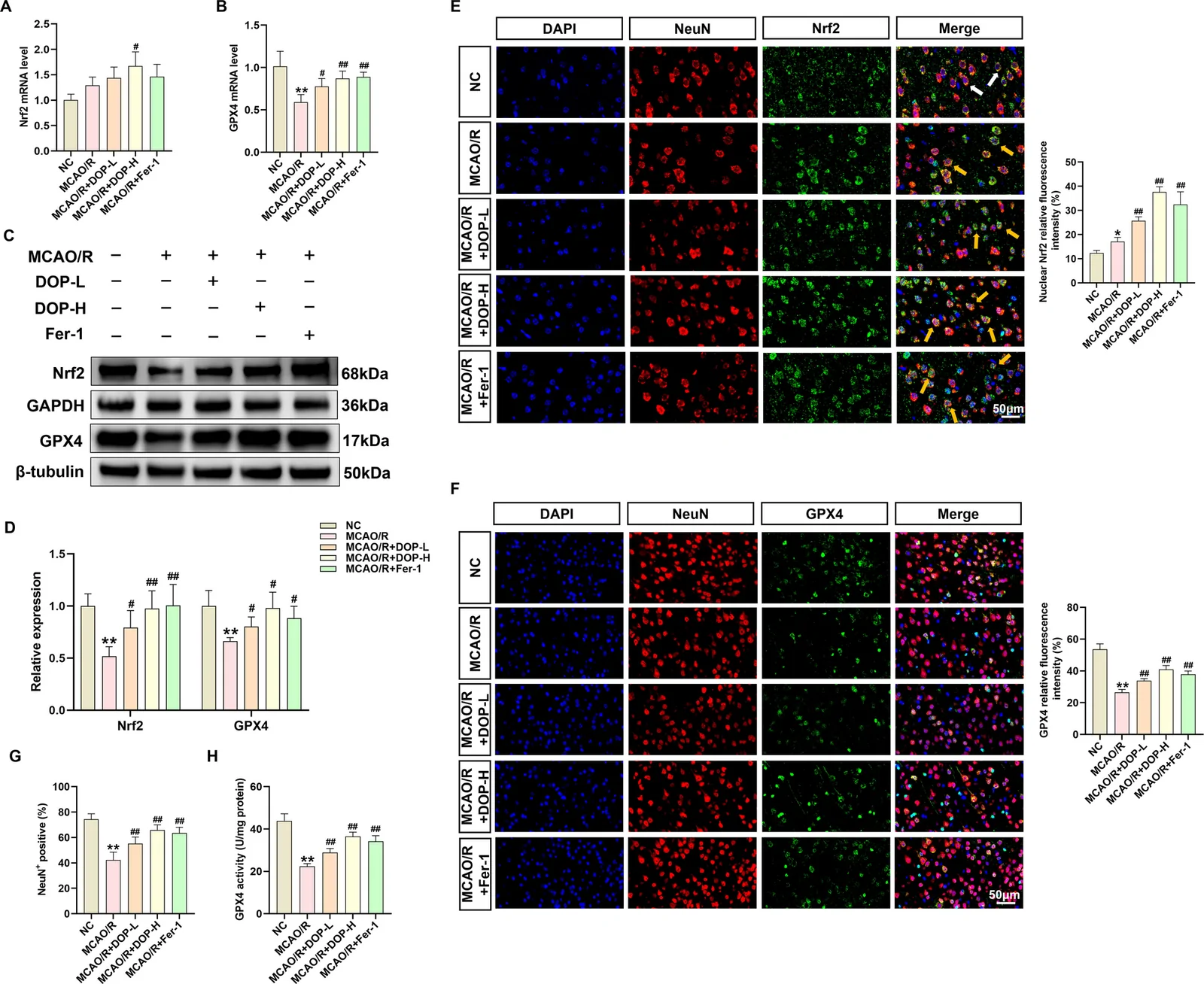
DOP promotes Nrf2 nuclear translocation and restores neuronal GPX4 expression in the MCAO/R model mice. **A** *Nrf2* mRNA expression measured by RT-qPCR. *n* = 6/group. **B** *GPX4* mRNA expression measured by RT-qPCR. *n* = 6/group. **C** Representative Western blot images of Nrf2 and GPX4 protein expression. *n* = 6/group. **D** Quantitative analysis of Nrf2 and GPX4 protein levels. *n* = 6/group. **E** Immunofluorescence staining of neurons (red) and Nrf2 (green) in the ischemic cortex. White arrows indicate cytoplasmic Nrf2; yellow arrows indicate nuclear-translocated Nrf2. Scale bar = 50 μm. *n* = 6/group. **F** Immunofluorescence staining of neurons (red) and GPX4 (green). Scale bar = 50 μm. *n* = 6/group. **G** Quantitative analysis of neuronal integrity marker NeuN fluorescence signals. *n* = 6/group. **H** GPX4 activity assay. *n* = 6/group. ^*^P < 0.05, ^**^P < 0.01 vs. NC group; ^#^*P* < 0.05, ^##^*P* < 0.01 vs. MCAO/R group

To investigate whether the neuroprotective effect of DOP is associated with ferroptosis regulation, activation of the Nrf2/GPX4 pathway and neuronal injury were assessed in MCAO/R mice. RT-qPCR and Western blot analyses showed that MCAO/R significantly reduced Nrf2 and GPX4 expression, whereas DOP treatment restored both mRNA and protein levels. Similar effects were observed in the MCAO/R + Fer-1 group (Fig. 4A–D). Immunofluorescence analysis further demonstrated increased neuronal Nrf2 nuclear localization and enhanced GPX4 fluorescence following DOP treatment compared with the MCAO/R group (Fig. 4E–F). Similar changes were observed after Fer-1 intervention. To evaluate neuronal injury, NeuN immunofluorescence staining was performed. MCAO/R markedly reduced NeuN-positive neuronal signals, whereas DOP and Fer-1 treatment restored NeuN fluorescence intensity, indicating improved neuronal survival after ischemia/reperfusion injury (Fig. 4G). Consistent with these findings, GPX4 activity was significantly decreased after MCAO/R but was restored by DOP and Fer-1 treatment (Fig. 4H).

Collectively, these results suggest that DOP alleviates neuronal injury and attenuates ferroptosis in MCAO/R mice, which may be associated with activation of the Nrf2/GPX4 signaling pathway.

### DOP Ameliorates OGD/R-induced Injury in HT-22 Cells

**Fig. 5:**
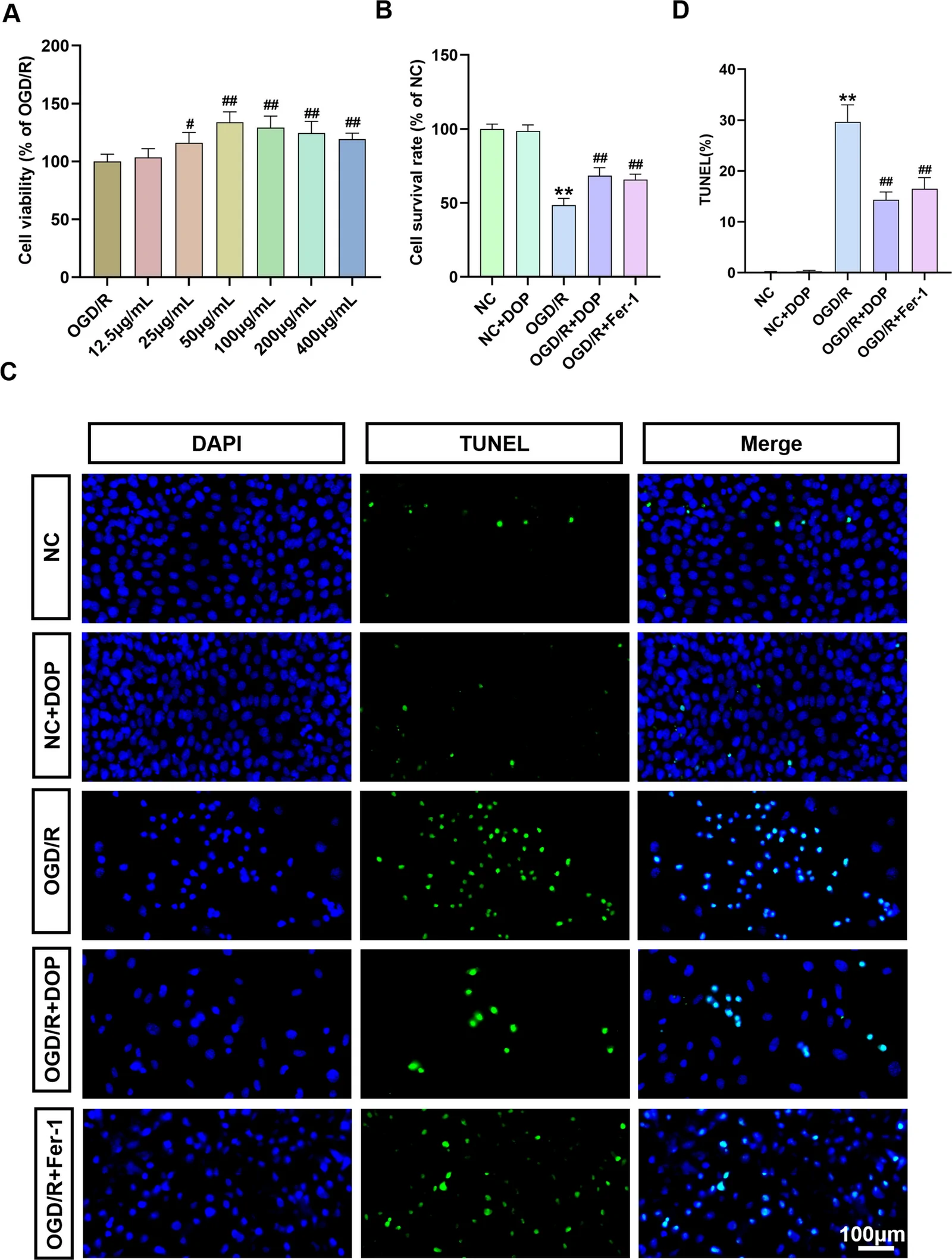
DOP ameliorates OGD/R-induced injury in HT-22 cells. **A** Viability of HT-22 cells treated with different concentrations of DOP, assessed by the CCK-8 assay. *n* = 6/group. **B** Cell survival rate measured by the CCK-8 assay. *n* = 6/group. **C** TUNEL staining of HT-22 cells. Scale bar = 100 μm, *n* = 3/group. **D** Percentage of TUNEL-positive cells. *n* = 3/group. ^**^P < 0.01 vs. NC group; ^#^*P* < 0.05, ^##^*P* < 0.01 vs. OGD/R group

To evaluate the effect of DOP on CI/RI in vitro, HT-22 cells were pretreated with DOP and subjected to OGD/R. CCK-8 assays demonstrated that DOP significantly improved cell viability in a concentration-dependent manner within the tested range (Fig. 5A). Notably, 50 μg/mL exhibited a pronounced protective effect without observable cytotoxicity and was therefore selected for subsequent experiments. Increasing the concentration beyond 50 μg/mL did not result in further significant improvement and was thus not pursued in subsequent analyses. Further analyses via CCK-8 and TUNEL staining revealed that OGD/R significantly reduced HT-22 cell survival and increased apoptosis. Both DOP and Fer-1 treatments notably enhanced cell survival and decreased apoptotic cell death (Fig. 5B–D). These results demonstrate that DOP attenuates OGD/R-induced injury in HT-22 cells.

### DOP Attenuates OGD/R-induced Ferroptosis in HT-22 Cells

**Fig. 6:**
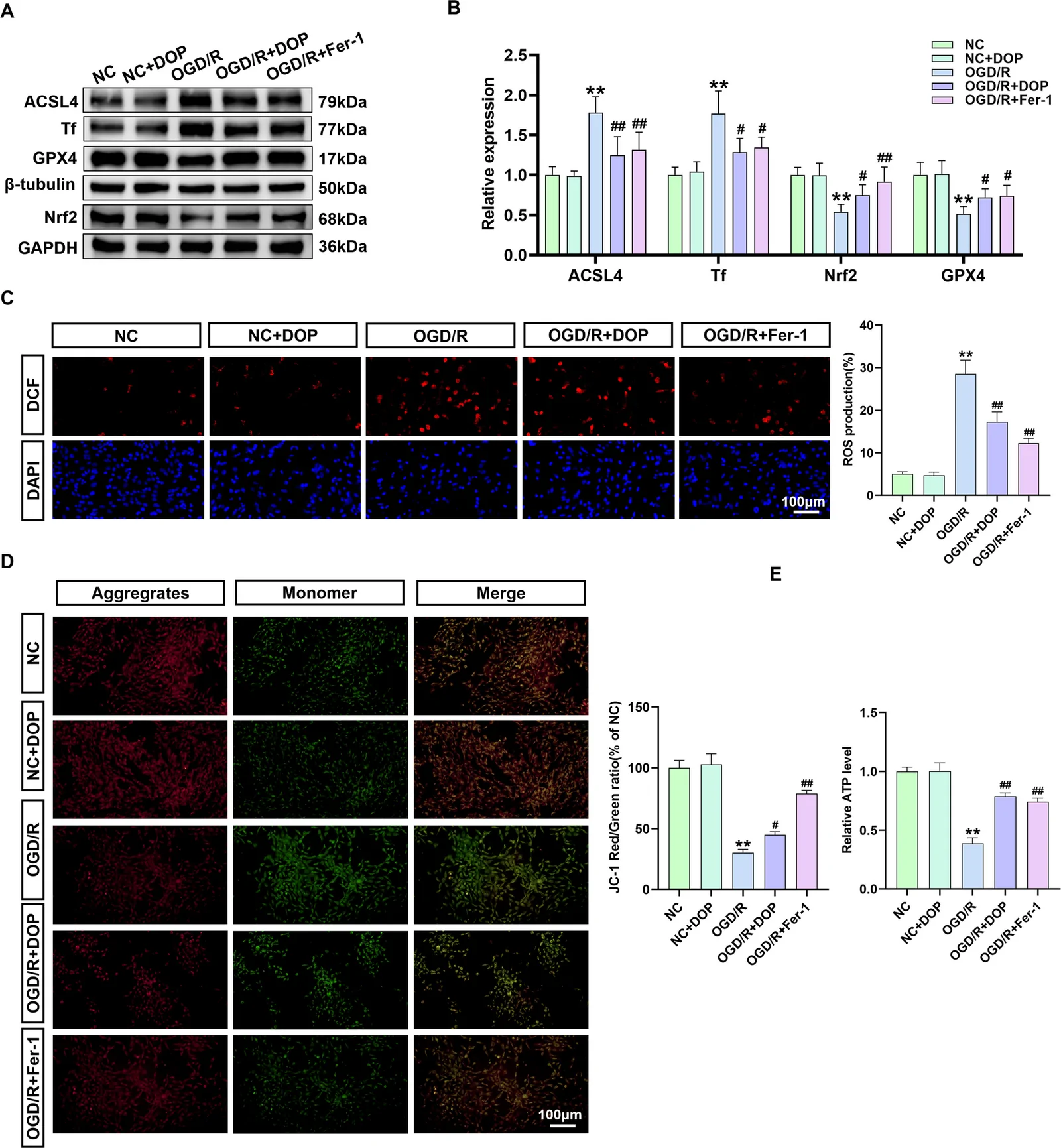
DOP attenuates OGD/R-induced ferroptosis in HT-22 cells. **A** Western blot analysis of ACSL4, Tf, Nrf2, and GPX4 protein expression. *n* = 6/group. **B** Quantitative analysis of protein expression levels. *n* = 6/group. **C** Representative fluorescence images of ROS staining. Scale bar = 100 μm, *n* = 3/group. **D** JC-1 fluorescence images showing MMP changes. Scale bar = 100 μm, *n* = 3/group. **E** ATP levels. *n* = 6/group. ^*^P < 0.05, ^**^P < 0.01 vs. NC group; ^#^*P* < 0.05, ^##^*P* < 0.01 vs. OGD/R group

To evaluate ferroptosis in HT-22 cells, we measured the expression of ferroptosis-related proteins, ROS levels, mitochondrial membrane potential (MMP), and ATP production. The results showed that OGD/R upregulated ACSL4 and Tf expression while downregulating Nrf2 and GPX4 levels (Fig. 6A–B), accompanied by increased ROS production, decreased MMP, and reduced ATP generation (Fig. 6C–E). In contrast, both DOP and Fer-1 treatments markedly reversed these alterations. These findings suggest that DOP attenuates OGD/R-induced ferroptosis in HT-22 cells, and the similar protective effects observed with Fer-1 further support the involvement of ferroptosis inhibition in this process.

### Nrf2/GPX4 Signaling Contributes to the Anti-ferroptotic Effects of DOP in OGD/R-Treated HT-22 Cells

**Fig. 7:**
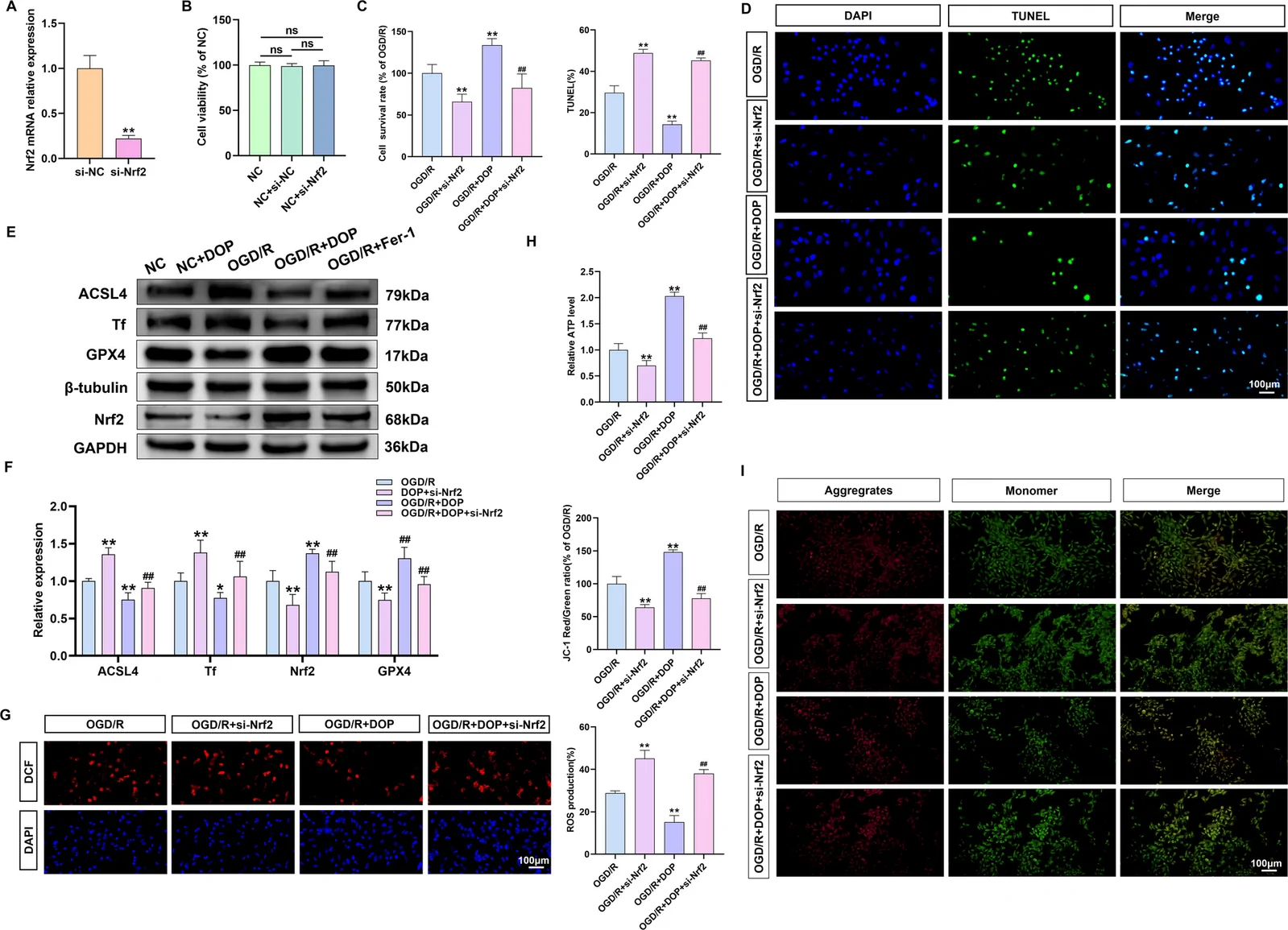
Nrf2/GPX4 signaling contributes to the anti-ferroptotic effects of DOP in OGD/R-treated HT-22 cells. **A** *Nrf2* mRNA expression detected by RT-qPCR. *n* = 3/group. **B**-**C** The CCK-8 assay assessed cell viability. *n* = 6/group. **D** TUNEL staining. Scale bar = 100 μm, *n* = 3/group. **E** Western blot analysis of ACSL4, Tf, Nrf2, and GPX4 protein expression. *n* = 6/group. **F** Quantitative analysis of protein expression levels. *n* = 6/group. **G** ROS fluorescence imaging. Scale bar = 100 μm, *n* = 3/group. **H** ATP levels. *n* = 6/group. **I** JC-1 staining, which shows the mitochondrial membrane potential. Scale bar = 100 μm, *n* = 3/group. ^*^P < 0.05, ^**^P < 0.01 vs. OGD/R group; ^#^*P* < 0.05, ^##^*P* < 0.01 vs. OGD/R + DOP group

To further investigate the role of the Nrf2/GPX4 pathway in the DOP-mediated suppression of ferroptosis after OGD/R, siRNA-mediated knockdown of Nrf2 was performed in HT-22 cells, and the transfection efficiency is shown in Fig. 7A. To determine whether Nrf2 knockdown exerts independent effects under basal conditions, additional control groups (NC + si-NC and NC + si-Nrf2) were included. Cell viability assays showed no significant differences among the NC, NC + si-NC, and NC + si-Nrf2 groups, indicating that Nrf2 knockdown alone does not affect basal cell viability under normoxic conditions (Fig. 7B). The results demonstrated that Nrf2 knockdown attenuated the protective and anti-ferroptosis effects of DOP, as evidenced by reduced cell survival and increased apoptosis (Fig. 7C–D). Moreover, Nrf2 knockdown led to elevated ROS levels, decreased MMP, and reduced ATP production, accompanied by downregulation of Nrf2 and GPX4 expression and upregulation of ACSL4 and Tf (Fig. 7E–I). These findings suggest that Nrf2 has a limited role under basal conditions but contributes to the cellular response to OGD/R-induced stress. Importantly, the protective effects of DOP against OGD/R-induced ferroptosis were partially attenuated by Nrf2 knockdown, suggesting that Nrf2/GPX4 signaling contributes, at least in part, to the anti-ferroptotic effects of DOP in HT-22 cells.

## Discussion

Recent studies have highlighted that ferroptosis is increasingly recognized as an important contributor to neuronal injury after ischemic stroke. Beyond iron accumulation and lipid peroxidation, emerging evidence indicates that ferroptosis is closely linked to metabolic reprogramming, including glycolytic dysregulation and lipid metabolic disturbances, as well as post-translational regulation such as ubiquitination-dependent control of ferroptosis-related proteins [33–35]. Consistent with these advances, our findings suggest that DOP alleviates CI/RI, at least in part, by suppressing ferroptotic responses, potentially through restoration of redox homeostasis and involvement of the Nrf2/GPX4 antioxidant pathway.

Ferroptosis is characterized by iron-dependent lipid peroxidation and disruption of cellular redox homeostasis [36]. Excess iron promotes ROS generation via the Fenton reaction, leading to lipid peroxidation and membrane damage. In the present study, we observed increased Fe^2^⁺ levels, decreased antioxidant capacity (SOD activity and GSH/GSSG ratio), and elevated lipid peroxidation markers (MDA, ACSL4, 4-HNE, and Tf) in MCAO/R mice, indicating ferroptosis injury. Consistently, OGD/R induced oxidative stress, mitochondrial dysfunction, and upregulation of ferroptosis-related markers in HT-22 cells. Ultrastructural mitochondrial damage further supported the occurrence of ferroptosis, highlighting its involvement in CI/RI pathology.

*Dendrobium officinale* polysaccharide (DOP) exhibits antioxidant, anti-inflammatory, and neuroprotective activities [20]. Consistent with previous reports [37], DOP significantly improved neurological deficits and reduced neuronal injury in MCAO/R mice, while also alleviating OGD/R-induced injury in HT-22 cells. Although anti-ferroptotic effects have been reported for *Dendrobium nobile* polysaccharides [18, 19], these findings cannot be directly extrapolated to *Dendrobium officinale*. We therefore investigated the role of ferroptosis in the neuroprotective effects of DOP. DOP attenuated ferroptosis-associated alterations both in vivo and in vitro, as evidenced by improved redox homeostasis, reduced lipid peroxidation, and decreased expression of ferroptosis-related proteins. DOP also ameliorated mitochondrial structural and functional abnormalities, including mitochondrial damage, loss of membrane potential, and ATP depletion. These findings suggest that suppression of ferroptosis and preservation of mitochondrial integrity may contribute to the protective effects of DOP against CI/RI.

The Nrf2/GPX4 signaling pathway is an important regulator of ferroptosis by maintaining cellular antioxidant defense and lipid peroxide homeostasis [38, 39]. Under oxidative stress conditions, Nrf2 activation promotes the expression of antioxidant and cytoprotective genes and has been implicated in the regulation of ferroptosis-associated molecules, including GPX4, SLC7A11, HO-1, and NQO1, thereby limiting ferroptotic damage [29, 40]. In this study, DOP enhanced neuronal Nrf2 nuclear localization and increased GPX4 expression both in vivo and in vitro. Furthermore, the restoration of GPX4 enzymatic activity following DOP treatment provided functional evidence supporting ferroptosis inhibition. Importantly, Nrf2 knockdown partially attenuated the protective and anti-ferroptotic effects of DOP, suggesting that Nrf2 signaling contributes to DOP-mediated neuroprotection. However, given the broad transcriptional regulatory functions of Nrf2, GPX4 may represent one of several downstream effectors involved in this process, and the precise molecular relationship between Nrf2 activation and GPX4 regulation requires further investigation.

Several limitations should be acknowledged. First, the current study employed a 30-day DOP pretreatment paradigm followed by short-term administration after reperfusion, which primarily reflects preventive or early intervention effects rather than a clinically applicable post-stroke therapeutic strategy. Whether DOP remains effective when administered after ischemic onset requires further investigation. Second, the pharmacokinetic and pharmacodynamic properties of DOP remain insufficiently characterized. Due to the structural complexity of polysaccharides, future studies should clarify its absorption, metabolism, active components, and potential ability to cross the blood–brain barrier. Third, although Nrf2 knockdown experiments support the involvement of Nrf2 signaling, in vivo genetic validation and GPX4 rescue approaches are still required to establish a more definitive causal relationship. Moreover, although immunofluorescence analysis supported enhanced neuronal Nrf2 localization and GPX4 expression, further quantitative approaches, such as nuclear-cytoplasmic fractionation or co-localization analysis, would provide additional evidence for Nrf2 activation. Finally, ferroptosis interacts with other regulated cell death pathways, including apoptosis, pyroptosis, and parthanatos, and the contribution of these pathways to DOP-mediated neuroprotection warrants further investigation.

## Conclusion

**Fig. 8:**
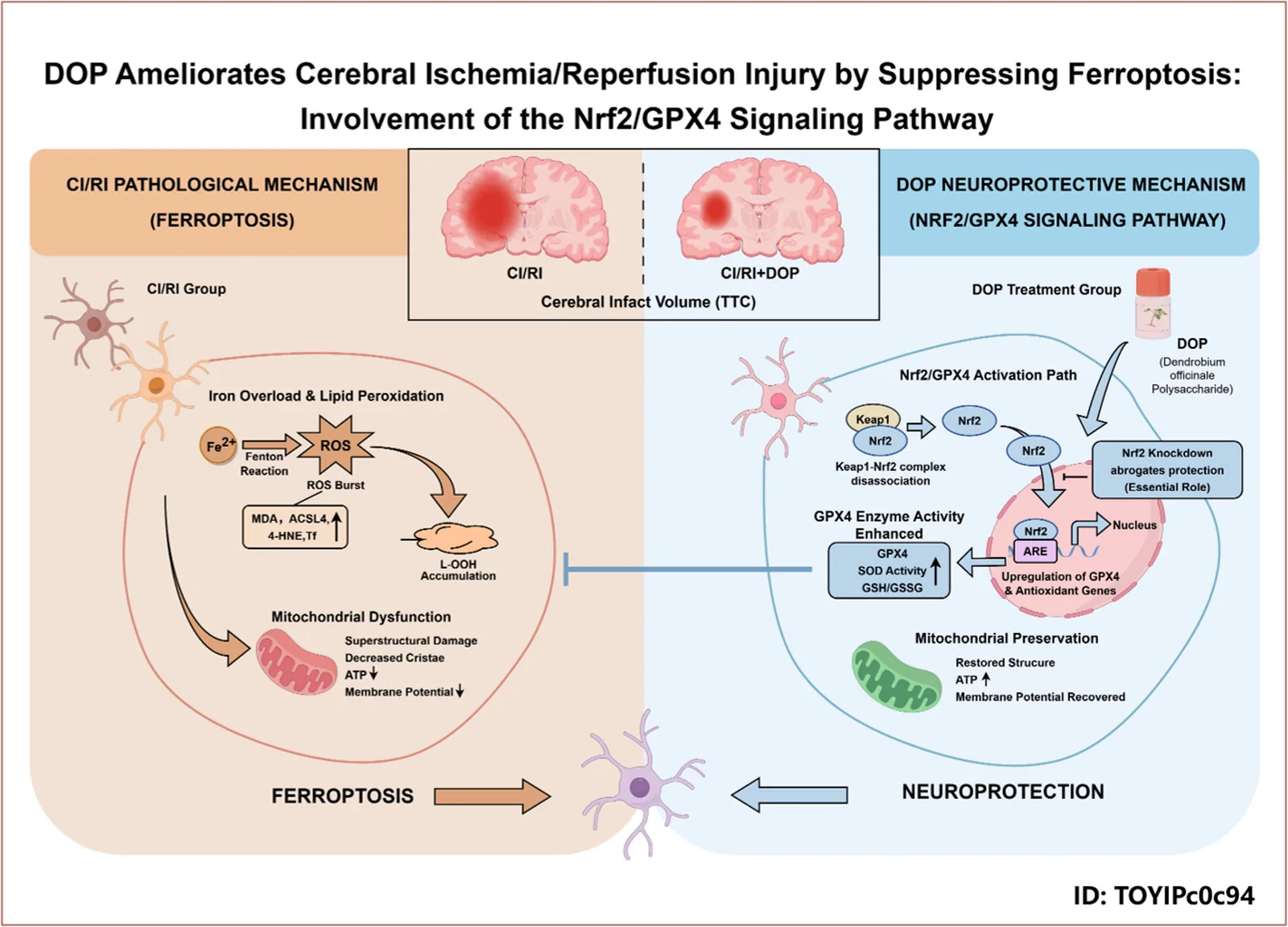
Proposed schematic model illustrating the neuroprotective mechanism of DOP against cerebral ischemia/reperfusion injury (By Figdraw). DOP exerts protective effects by modulating ferroptosis-related processes, including the regulation of iron homeostasis, inhibition of lipid peroxidation, and preservation of mitochondrial function. Mechanistically, these effects are associated, at least in part, with activation of the Nrf2/GPX4 signaling pathway. Through these coordinated actions, DOP mitigates neuronal injury and contributes to neuroprotection during ischemia/reperfusion, supporting its potential role in preventive and early-stage therapeutic strategies for ischemic stroke

In conclusion, this study demonstrates that DOP exerts neuroprotective effects against cerebral ischemia/reperfusion injury. These effects are associated, at least in part, with suppression of ferroptosis-related processes and involvement of the Nrf2/GPX4 signaling pathway (Fig. 8). Our findings provide mechanistic insights into the neuroprotective properties of DOP and suggest its potential relevance as a preventive or early-stage intervention strategy for ischemic stroke. Further studies using clinically relevant post-stroke treatment paradigms, pharmacokinetic characterization, and in vivo pathway validation are warranted to further evaluate its translational potential.

## Data Availability

All data generated or analysed during this study are included in this published article.
